# The Hepatitis C Virus Modulates Insulin Signaling Pathway *In Vitro* Promoting Insulin Resistance

**DOI:** 10.1371/journal.pone.0047904

**Published:** 2012-10-25

**Authors:** José A. del Campo, Marta García-Valdecasas, Lourdes Rojas, Ángela Rojas, Manuel Romero-Gómez

**Affiliations:** Unit for Medical and Surgical Management of Digestive Diseases and CIBERehd, Valme University Hospital, Sevilla, Spain; Institut Pasteur, France

## Abstract

Insulin is critical for controlling energy functions including glucose and lipid metabolism. Insulin resistance seems to interact with hepatitis C promoting fibrosis progression and impairing sustained virological response to peginterferon and ribavirin. The main aim was to elucidate the direct effect of hepatitis C virus (HCV) infection on insulin signaling both *in vitro* analyzing gene expression and protein abundance. Huh7.5 cells and JFH-1 viral particles were used for in vitro studies. Experiments were conducted by triplicate in control cells and infected cells. Genes and proteins involved in insulin signaling pathway were modified by HCV infection. Moreover, metformin treatment increased gene expression of PI3K, IRS1, MAP3K, AKT and PTEN more than >1.5 fold. PTP1B, encoding a tyrosin phosphatase, was found highly induced (>3 fold) in infected cells treated with metformin. However, PTP1B protein expression was reduced in metformin treated cells after JFH1 infection. Other proteins related to insulin pathway like Akt, PTEN and phosphorylated MTOR were also found down-regulated. Viral replication was inhibited in vitro by metformin. A strong effect of HCV infection on insulin pathway-related gene and protein expression was found in vitro. These results could lead to the identification of new therapeutic targets in HCV infection and its co-morbidities.

## Introduction

Some metabolic disorders including obesity, steatosis and insulin resistance are known to play a major role in the response to peginterferon/ribavirin and fibrosis progression in patients with chronic hepatitis C [1], [2]. Molecular, pathological, epidemiological, randomized controlled trials and observational studies have highlighted the relationship between hepatitis C virus and glucose metabolism [3], [4]. However, the effect of adding insulin sensitizers (like metformin or pioglitazone) to peginterferon plus ribavirin remain controversial [5], [6]. There is growing evidence that metabolic perturbations associated with HCV infection may result from interactions between viral and host proteins [7], [8].The insulin receptor belongs to a subfamily of receptor tyrosine kinases that includes the IGF (Insulin-like Growth Factor) receptor and the IRR (Insulin Receptor-Related Receptor) [9]. Insulin has diverse effects on cells including stimulation of glucose transport, gene expression and alterations of cell morphology. These effects utilize different signaling pathways: i) adaptor molecules such the IRS (Insulin Receptor Substrates), the SHC (Src and Collagen Homologues) and the GRB2 (Growth Factor Receptor Binding protein-2), ii) lipid kinases such as PI3K (Phosphatidylinositol 3-Kinase), iii) small G-proteins like Rac, and iv) serine, threonine and tyrosine kinases [10]. Proteins involved in insulin signaling display binding sites for numerous signaling partners from Akt inhibiting apoptosis by phosphorylating the BAD (BCL2 Antagonist of Cell Death) component of the BAD/BCLXL complex [11] or activating mTOR (Mammalian Target of Rapamycin)/FRAP pathway to protein tyrosine phosphatase (PTPase) [12] (Protein Tyrosine Phosphatases) that catalyzes the dephosphorylation of insulin receptor and its substrates, leading to attenuation of insulin action.

**Figure 1:**
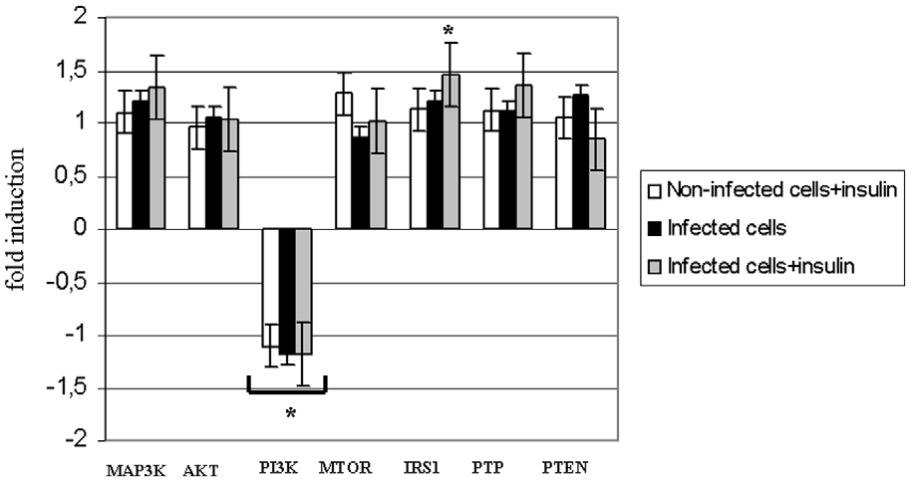
Insulin effect on gene expression in vitro. Relative gene expression (fold induction [+] and fold inhibition [−]) in Huh7.5 cells grown in the presence of insulin (10 nM) and JFH-1 particles (one particle per cell). Experiments were performed in triplicate. Control experiment (non-infected cells and no insulin added) was used as a reference (fold induction = 1). (*) = p<0.05.

PTP1B has been shown to function as the insulin receptor phosphatase [13]. Insulin stimulates cell growth and differentiation, and promotes the storage of substrates in fat, liver and muscle by stimulating lipogenesis, glycogen and protein synthesis, and inhibiting lipolysis, glycogenolysis and protein breakdown. Insulin resistance or deficiency results in profound dysregulation of these processes, and produces elevations in fasting and postprandial glucose and lipid levels.

Metformin improves insulin sensitivity, inhibits hepatic gluconeogenesis and decreases glycogenolysis. It is an activator of AMP-activated protein kinase (AMPK) signalling, [14], [15] and reduces mTOR pathway. Metformin also inhibits cancer cell growth by inducing cell cycle arrest and enhancing apoptosis [16]. A controlled, randomized, double-blind clinical trial (TRIC-1) examined the effect of adding metformin to standard therapy in the treatment of hepatitis C [4]. This study demonstrated that women infected with hepatitis C virus genotype 1 and HOMA >2 treated with metformin showed a greater drop in viral load during the first 12 weeks and a doubled sustained viral response in comparison with females receiving placebo. Recently, García-Ruiz et al. [17] have demonstrated that inhibition of PTP1B using pervanadate restores insulin and interferon response. These authors have found that metformin is able to reduce PTP activity.

We aim to analyze in vitro the effect of HCV infection on insulin signaling pathway elements involving gene and protein expression. This aim was partially achieved by the identification of key elements involved in HCV-related insulin resistance response (like PTP1B). Interestingly, metformin was found to inhibit viral replication in vitro.

**Figure 2:**
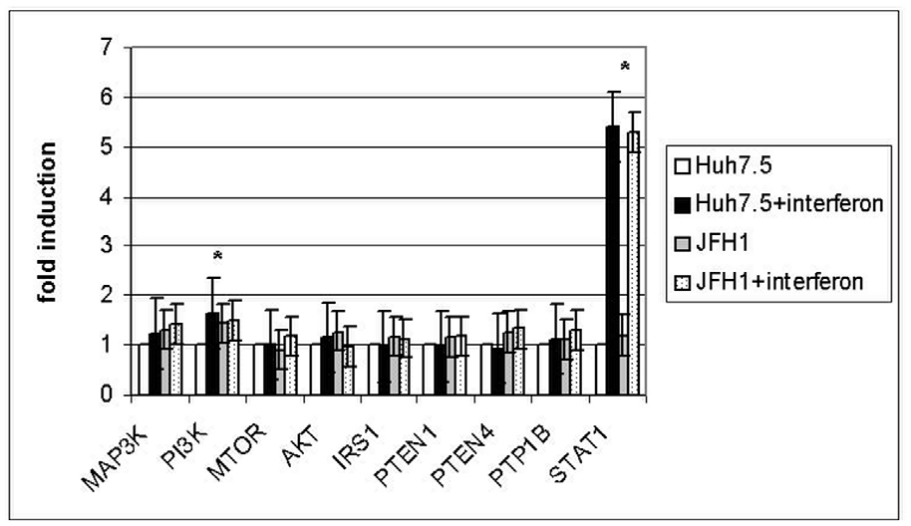
Interferon effect on insulin pathway gene expression. Huh7.5 cultures were treated with 500 IU/ml and infected with JFH-1 particles. Experiments were performed in triplicate. Control experiment (non-infected cells and no insulin added) was used as a reference (fold induction = 1). (*) = p<0.05.

## Materials and Methods

### Cell Culture and Gene Expression Assays

Huh7.5 cells (Apath LLC, New York, USA) were grown in DMEM culture medium supplemented with 10%FBS, antibiotics, L-Glutamine and Non-Essential aminoacids. Cells were incubated at 37°C, 5%CO_2_. Cell culture-derived virus particles JFH-1, were generated as previously described [18]. Infective particles of JFH-1 were added to growing cells at 1 particle/cell rate. In general, infective particles were added 24 hours after cell seeding, incubated together with the cells for 48 hours. Then, cultured media was removed and fresh virus-free media was added to cell cultures and incubated for additional 48 hours. Total RNA was extracted from cellular lysates using standard protocols. We have performed the respective retro-transcription reactions using commercially available kits (Qiagen, Invitrogen, Carlsbad, CA, USA). Gene expression was analyzed by semi-quantitative real-time PCR using a Stratagene model MX3005P cycler. Insulin (10 nM) purchased from Sigma-Aldrich (St. Louis, USA), metformin (2 mM) purchased from Acofarma (Barcelona, Spain) and α-interferon (500 IU/ml) purchased from Sigma-Aldrich (St. Louis, USA) were added to culture media when indicated.

**Figure 3:**
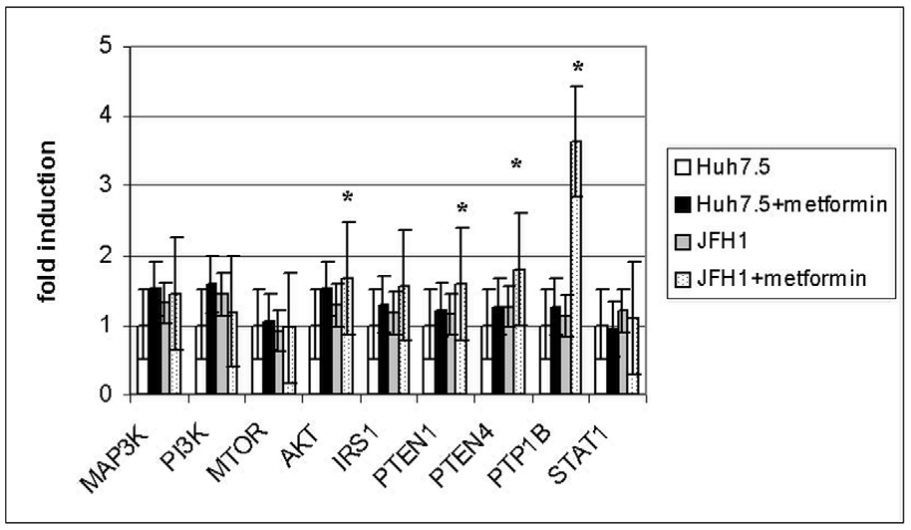
PTP1B is highly induced in the presence of metformin and HCV. Gene expression (fold induction) of insulin pathway-related genes in Huh7.5 cells treated with metformin (2 mM) was analyzed. Experiments were performed in triplicate. (*) = p<0.05.

### JFH-1 Replication Analysis

Primers sequences used for JFH1 replication were: fwd- CTGTGAGGAACTACTGTCT and reverse: CGCCCTATCAGGCAGTACCA. All experiments involving JFH-1 infective particles have been performed in triplicate in a P3 laboratory. The presence of JFH-1 RNA in cell cultures was determined by RT-PCR using specific primers.

**Figure 4:**
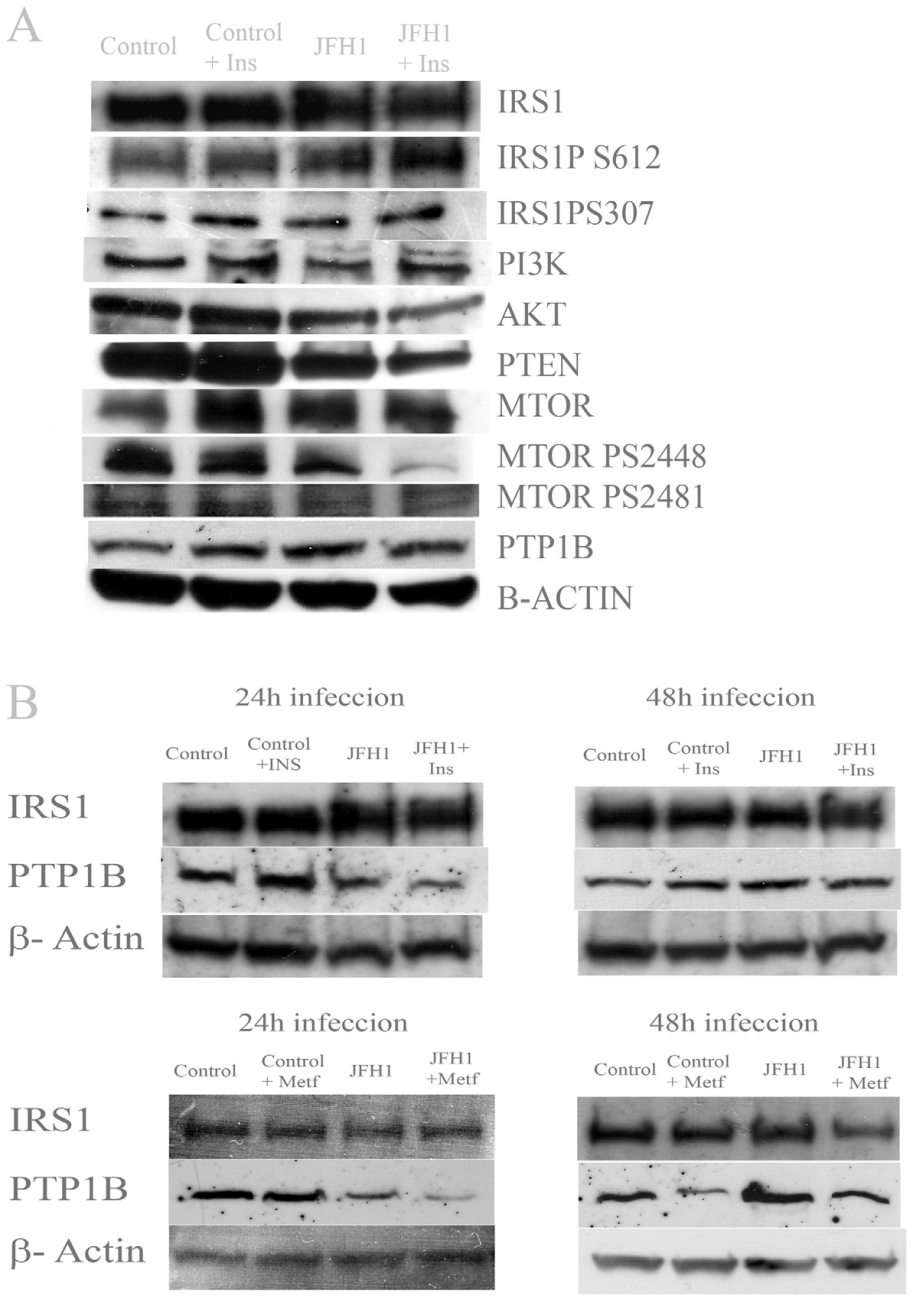
Insulin pathway proteins are down-regulated by JFH1. **A**: Western blot from Huh7.5 cells in the presence or absence of insulin (20 nM) and infected or not with JFH-1 particles. 40 µgrams of total protein were loaded per lane. A representative gel is shown for each protein. All experiments were performed in triplicate. β-Actin was used as a loading control. **B**: IRS1 and PTP1B abundance at 24 and 48 hours post-infection. Huh7.5 cells were infected with JFH1 particles and incubated at the indicated time. Performance of western-blot was as indicated for panel **A**.

### Protein Analysis

Cells were disrupted using a commercial kit for protein extraction (Qiagen, Hilden, Germany) and proteins were quantified using Bradford method. For western-blot analysis, total protein (40 µg) were loaded onto 10–12% SDS-PAGE. Primary antibodies (IRS1, IRS1-P, IRS1-P S612, IRS1-P S636/639, mTOR, mTOR-P S2448, MTOR-P S2481, PTEN-1, PTP1B, and β-ACTIN (for loading control) were purchased from Cell Signaling Technology (Beverly, MA, USA).

### Statistical Analysis

Continuous variables were summarized as means ± SD and categorical variables as frequency and percentage. Comparisons between groups were made by using the Student t test or the Mann-Whitney U test for continuous variables and the [chi]^2^ test or Fisher exact probability test for categorical data. Two-sided P values <0.05 were considered statistically significant. Data were collected in a computerized database and analyzed using the SPSS package (SPSS 18.0 for Windows, Chicago, IL).

**Figure 5:**
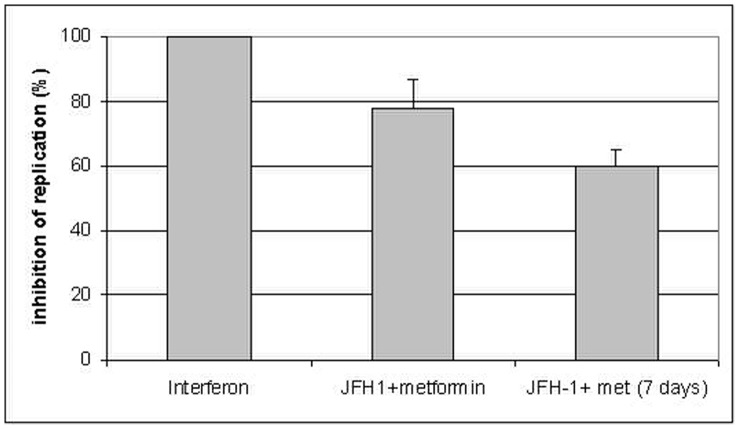
Metformin (2 mM) inhibits viral replication in vitro. Huh7.5 cells treated with interferon (500 IU/ml) were used as a positive control for inhibition of viral replication (100%). Cells were infected with JFH1 and treated with metformin for 48 hours and 7 days. Metformin significantly inhibited viral replication (p<0.05).

## Results

### Insulin Signaling-related Gene Expression in vitro

Figure 1 shows relative gene expression (relative fold induction) in the presence or absence of insulin (10 nM) in both control and infected cells. Significant differences were found for IRS1 fold induction (1.5±0.23) and PI3K fold inhibition (−1.2±0.3) (p<0.05). Since all anti-HCV therapies are based on α-interferon, we further investigate the role of this molecule in insulin pathway-gene expression (Figure 2). As expected, interferon treatment promoted STAT1 gene expression induction (>5 fold). PI3K gene expression was also significantly induced in the presence of interferon. Other genes related to the insulin signaling pathway were not found to be altered in their expression pattern (Figure 2).

### Metformin Modifies Insulin-related Gene Expression

We previously shown that adding metformin to peginterferon and ribavirin was safe and improved insulin sensitivity in chronic hepatitis C patients [4]. To further investigate the mechanism involved in this process, Huh7.5 cells were grown in the presence of metformin and its effect on gene expression was analyzed. Results are shown on Figure 3. Most of the genes involved in insulin pathway (PI3K, IRS1, MAP3K, AKT, PTEN and PTP1B) are induced in the presence of metformin. AKT, PTEN and PTP1B were induced higher than 1.5-fold (p<0.05). This effect is particulary evident in the presence of JFH-1 particles. PTP1B, encoding a tyrosin phosphatase which plays an important role in insulin signaling, is highly induced in the presence of metformin and HCV.

### Protein Expression in Huh7.5 Cells

Total protein extracts were obtained from Huh7.5 cells treated with insulin and infected with JFH-1 particles and protein abundance was analyzed by inmmunoblot. Results are shown on Figure 4A and 4B. Major changes in protein expression were observed in AKT, PTEN and phosphorylated MTOR PS2448. These proteins were found down-regulated in the presence of JFH-1 particles and insulin. A representative western-blot is shown on Figure 4. All experiments were performed in triplicate, JFH1 particles were added to Huh7.5 cell cultures and incubated for 48 h. PTP1B protein was found down-regulated after 24 hours of infection (Fig. 4B) when cells were treated either with insulin or metformin. However, this effect was not observed after 48 hours of infection. IRS1 protein was reduced after 48 hours of infection in the presence of metformin.

### Metformin Inhibits Viral Replication in vitro

Since metformin affects gene and protein expression in Huh7.5 cells, we wonder if this compound could exert any effect on viral replication. JFH-1 RNA was determined in total RNA extracted from Huh7.5 cells in the presence of metformin (2 mM) compared to cells grown with α-interferon (500 IU/ml). Results (Figure 5) shown that metformin could inhibit significantly viral replication (∼80%) compared to interferon (100%) (p<0.05). These experiments were performed in our usual conditions (see Methods for details). When cells were incubated for longer periods (after one week) viral replication was inhibited by 60% (Figure 5).

## Discussion

In this work, it has been demonstrated that HCV interacts in vitro, with insulin pathway affecting gene expression in vitro and in vivo trough different mechanisms: it modifies insulin pathway-related gene and protein expression in Huh7.5 cells in response to treatment (insulin, α-interferon and metformin On the other hand, metformin inhibited viral replication in vitro, where PTP1B seems to play an essential role.

These results are also specially relevant for PI3K gene since the presence of metformin in the media enhances this effect not only for PI3K but also for IRS1, MAP3K, AKT and PTEN. PI3K activates AKT and this serine/threonine kinase phosphorylates FoxO1. It is well established that this transcription factor mediates the expression of genes involved in both glucose and lipid metabolism [19]–[22].

Protein tyrosine phosphatase 1B (PTP1B) plays important roles in down-regulation of insulin and leptin signaling and is an established therapeutic target for diabetes and obesity [17], [23]. PTP1B is regulated by reactive oxygen species (ROS) produced in response to various stimuli, including insulin. It has been shown that PTP1B inhibitor improves insulin resistance and lipid abnormalities in vitro and in vivo [24]. We have shown that PTP1B protein is reduced 24 hours post-infection in cells treated with insulin and metformin (see Fig. 4B). Down-regulation of this protein could explain the reduction of IRS1 in the presence of metformin. This effect on total IRS1 is not observed in the presence of insulin, but phosphorylated IRS1 protein (S612) is increased after 48 hours post-infection (Fig. 4A). PTP1B has a unique role in modulating the signaling of IRS1 and IRS2 in the regulation of hepatic metabolic pathways and represents a rational target for the development of new pharmacological agents aimed at improving hepatic insulin sensitivity [25]. Metformin treatment of infected cells promoted a strong effect on gene expression (PI3K, IRS1, MAP3K, AKT, PTEN) (Figure 3). These genes involved in the insulin signaling pathway were found to be significantly induced, and this effect was particularly relevant for PTP1B. This fact corroborates the role of this protein in the outcome of HCV-dependent insulin resistance. Recently, García-Ruiz et al. [17] have shown that inhibition of PTP-1B activity with pervanadate and metformin or knocking down PTP-1B reestablishes IFNα response in vitro. Likewise, metformin decreases PTP-1B activity and improves response to IFNα in insulin-resistant obese mice. Moreover, our results indicate that metformin inhibited about 80% JFH-1 replication in Huh7.5 cells when compared to interferon-a treatment, supporting the idea that promoting insulin resistance in HCV infection is highly relevant for viral replication [26]. This effect could be explained by the fact that metformin is an AMPK activator [27] and this protein inhibits mTOR. In this case, mTOR activity should be relevant for viral replication.

mTOR is an evolutionarily conserved serine/threonine protein kinase, and it functions as a central signaling molecule involved in a wide array of cellular processes, such as cell survival, proliferation, and metabolism. Peng et al. [28] have shown that HCV-encoded viral protein up-regulates the mTOR pathway to block apoptosis and imply that NS5A may contribute not only to HCV persistence but also to the development of HCV-related diseases. Protein analysis in this work show that mTOR abundance in Huh7.5 cells is lower in the presence of JFH-1 and insulin (Fig. 4), indicating an interaction between this protein and HCV proteins. On the other hand, mitogen-activated protein kinase (MAP3K) is up-regulated in adipose tissue of obese mice and patients and correlated with TNF-alpha expression [29], but according to our knowledge, there is no previous report on HCV infection or insulin resistance involving MAP3K.

Clèment et al. [30] showed that the core protein of HCV genotype 3a (but not 1b) induced a 50% decrease in the PTEN protein expression level in Huh-7 and HepG2 cells. They concluded that HCV core 3a modulates PTEN expression indirectly because no physical interactions of core 3a and PTEN were detected. The expression of PTEN in Huh7.5 cells infected with JFH1 did not change significantly (Figure 1) but the abundance of protein in infected cells was lower compared to uninfected cells. This effect was enhanced in the presence of insulin (Fig. 4A). A possible explanation would be the occurrence of post-transcriptional regulation events. Metformin improves insulin resistance through AMPK activation. However, when Huh7.5 cells are infected, PTEN (an insulin pathway inhibitor) gene expression is induced in the presence of metformin (Fig. 3). This fact is probably due to an interaction with viral proteins, promoting enhanced insulin resistance.

Taking all these evidences together we can conclude that HCV interacts with insulin signaling pathway through different mechanisms: it changes the expression of key genes in insulin metabolism (PI3K, PTEN, MAP3K and PTP1B).On the other hand, HCV modulates protein expression in vitro through down-regulation of PTP1B, PTEN and MTOR and when metformin is used, the viral replication is inhibited as shown on Fig. 5. These results could lead to the identification of new host related therapeutic targets (like PTP1B) in HCV infection.
